# Introducing *Palliative Medicine Reports*: Dedicated to Sharing Palliative Care Knowledge with the World

**DOI:** 10.1089/pmr.2020.28999.editorial

**Published:** 2020-04-30

**Authors:** Christopher A. Jones

**Affiliations:** Department of Medicine and Palliative and Advanced Illness Research Center, University of Pennsylvania, Philadelphia, Pennsylvania, USA.

It is with great excitement that we release the first articles from *Palliative Medicine Reports* (PMR) to the palliative care community. PMR is an open-access companion to the *Journal of Palliative Medicine* (JPM) that gives authors in palliative care and related fields from around the globe a forum to disseminate their work.

As palliative care has grown from a niche specialty to one that is integrated into most every facet of medical care, our workforce and literature base have also grown. What has expanded more slowly, though, are high-quality, reputable journals with thoughtful peer review that enable the sharing of the breadth of clinical research, case series, humanistic reflections, and opinion pieces in palliative care.

The *Journal of Palliative Medicine* receives almost 700 submissions per year but, as a paper journal, it lacks the space needed to print many articles worthy of publication. Unfortunately, this unmet need for avenues of scientific dissemination has led to an increase in predatory journals. Often lacking editorial boards and peer review, predatory journals have grown in number in the last decade and can trap authors' manuscripts and copyright until extortive fees are paid.^1^

It is our hope that PMR is well positioned to meet many of the currently unmet needs. An online-only publication, PMR is able to publish any well-done article that will interest those in our field. PMR will ensure the manuscripts published are of high quality and meet the peer- review standards of the shared group of JPM/PMR peer reviewers. Deeply appreciative of ongoing support and mentorship from the JPM Editorial Board and Managing Editor, PMR has begun to set its own course by assembling a cadre of enthusiastic Associate Editors from Duke University, the University of Nebraska, Penn State College of Medicine, Seattle Children's Hospital, the University of Rochester, Moffit Cancer Center, the University of Pennsylvania, and others. This group will work to promote PMR and support authors, guiding PMR toward its own niche in palliative care.

A major difference between PMR and most other academic journals centers around the publishing model. Most academic publications are free to authors at the point of publication but the resulting science is housed behind paywalls and copyright for articles is held by the publisher. The costs of publication are paid by readers, either by individual subscription, as a per-article charge, or by libraries and institutions with annual subscriptions. For those without access, especially true in resource-limited countries, accessing articles published in traditional journals can be impossible. PMR, by contrast, has adopted the “open access” model. After rigorous peer review and revision, accepted manuscripts will be assigned a Creative Commons CC-BY license to enable public sharing. Articles will be freely available because the cost of publishing is paid by authors on a sliding scale depending on the socio-economic status of the authors' country of origin. Many organizations that fund research, including the Bill and Melinda Gates Foundation, the Wellcome Trust, and nearly two dozen European funders, have come together to support “Plan S,” which “mandate[s] that access to research publications that are generated through research grants that they allocate must be fully and immediately open.”^2^

While different from the model many Americans are accustomed to, high-quality, peer-reviewed, open-access publishing is likely an important part of the future of academic publishing worldwide. *Palliative Medicine Reports* is proud to be at the vanguard of this movement. We at PMR are excited to support authors with clinical research, cases, position papers, reflections, and opinions to share as we make quality information in palliative care and related fields accessible to the world.

## Abbreviations Used

JPM: *Journal of Palliative Medicine*
PMR: Palliative Medicine Reports
